# Polygenic scores for risk of pancreatic ductal adenocarcinoma: evaluation of novel and published models

**DOI:** 10.1038/s41698-026-01479-x

**Published:** 2026-05-27

**Authors:** Samuel O. Antwi, Brandon J. Coombes, Erin E. Carlson, Nicholas B. Larson, Kari G. Rabe, Hunter J. Atkinson, Shounak Majumder, William R. Bamlet, Daniel J. Schaid, Jun Zhong, David McKean, Alan A. Arslan, Laura E. Beane Freeman, Paige M. Bracci, Federico Canzian, Thérèse Truong, Josh Atkins, Mengmeng Du, Steven Gallinger, Phyllis J. Goodman, Verena Katzke, Daniele Campa, Charles Kooperberg, Loic Le Marchand, Rachel E. Neale, Alpa V. Patel, Jean Wactawski-Wende, Sandra Perdomo, Xiao-Ou Shu, Kala Visvanathan, Stephen K. Van Den Eeden, Emily White, Wei Zheng, Demetrius Albanes, Gabriella Andreotti, Paul Brennan, Stephen J. Chanock, Yu Chen, Burcu Darst, Pietro Ferrari, Edward L. Giovannucci, Michael Goggins, Christopher Haiman, Manal Hassan, Rayjean J. Hung, Miranda R. Jones, Peter Kraft, Núria Malats, Steven C. Moore, Kimmie Ng, Ulrike Peters, Miquel Porta, Nathaniel Rothman, Maria-José Sánchez, Howard D. Sesso, Debra T. Silverman, Melissa C. Southey, Roger L. Milne, Caroline Y. Um, Herbert Yu, Chen Yuan, MAYO-RGC Project Generation, James R. Cerhan, Fergus J. Couch, Nicholas B. Larson, Eric W. Klee, Zachary S. Fredericksen, Mine Cicek, Janet E. Olson, Lisa K. Colborn, Andrew J. Danielsen, Jonathan J. Harrington, Regeneron Genetics Center, Aris Baras, Gonçalo Abecasis, Adolfo Ferrando, Giovanni Coppola, Andrew Deubler, Luca A. Lotta, John D. Overton, Jeffrey G. Reid, Alan Shuldiner, Katherine Siminovitch, Jason Portnoy, Marcus B. Jones, Lyndon Mitnaul, Alison Fenney, Jonathan Marchini, Manuel Allen Revez Ferreira, Maya Ghoussaini, Mona Nafde, William Salerno, Cristen Willer, Lourdes Crane, Christina Beechert, Erin Fuller, Laura M. Cremona, Eugene Kalyuskin, Hang Du, Caitlin Forsythe, Zhenhua Gu, Kristy Guevara, Michael Lattari, Alexander Lopez, Kia Manoochehri, Prathyusha Challa, Manasi Pradhan, Raymond Reynoso, Ricardo Schiavo, Maria Sotiropoulos Padilla, Chenggu Wang, Sarah E. Wolf, Manan Goyal, George Mitra, Sanjay Sreeram, Rouel Lanche, Vrushali Mahajan, Sai Lakshmi Vasireddy, Gisu Eom, Krishna Pawan Punuru, Sujit Gokhale, Benjamin Sultan, Pooja Mule, Mudasar Sarwar, Muhammad Aqeel, Xiaodong Bai, Lance Zhang, Sean O’Keeffe, Razvan Panea, Evan Edelstein, Ayesha Rasool, Evan K. Maxwell, Boris Boutkov, Alexander Gorovits, Ju Guan, Lukas Habegger, Alicia Hawes, Olga Krasheninina, Samantha Zarate, Adam J. Mansfield, Joshua Backman, Kathy Burch, Adrian Campos, Liron Ganel, Sheila Gaynor, Benjamin Geraghty, Arkopravo Ghosh, Salvador Romero Martinez, Christopher Gillies, Lauren Gurski, Eric Jorgenson, Tyler Joseph, Michael Kessler, Jack Kosmicki, Adam Locke, Priyanka Nakka, Karl Landheer, Olivier Delaneau, Anthony Marcketta, Joelle Mbatchou, Arden Moscati, Anita Pandit, Jonathan Ross, Carlo Sidore, Eli Stahl, Timothy Thornton, Sailaja Vedantam, Rujin Wang, Kuan-Han Wu, Bin Ye, Blair Zhang, Andrey Ziyatdinov, Yuxin Zou, Jingning Zhang, Kyoko Watanabe, Mira Tang, Frank Wendt, Suganthi Balasubramanian, Suying Bao, Kathie Sun, Chuanyi Zhang, Sean Yu, Aaron Zhang, David Corrigan, Dhruv Shidhaye, Chen Wang, Keyrun Adhikari, Alexander Lachmann, Brian Hobbs, Jon Silver, William Palmer, Rita Guerreiro, Amit Joshi, Antoine Baldassari, Sarah Graham, Ernst Mayerhofer, Erola Pairo Castineira, Mary Haas, Niek Verweij, George Hindy, Jonas Bovijn, Tanima De, Luanluan Sun, Olukayode Sosina, Arthur Gilly, Peter Dornbos, Juan Rodriguez-Flores, Moeen Riaz, Manav Kapoor, Gannie Tzoneva, Momodou W. Jallow, Anna Alkelai, Ariane Ayer, Veera Rajagopal, Sahar Gelfman, Vijay Kumar, Jacqueline Otto, Jose Bras, Silvia Alvarez, Jessie Brown, Hossein Khiabanian, Joana Revez, Kimberly Skead, Valentina Zavala, Jae Soon Sul, Lei Chen, Sam Choi, Amy Damask, Nan Lin, Charles Paulding, Sameer Malhotra, Joseph Herman, Michelle G. LeBlanc, Nadia Rana, Jennifer Rico-Varela, Jaimee Hernandez, Larizbeth Romero, Ashley Paynter, Randi Schwartz, Jody Hankins, Anna Han, Samuel Hart, Ryan Smith, Ann Perez-Beals, Gina Solari, Johannie Rivera-Picart, Michelle Pagan, Sunilbe Siceron, Harvey A. Risch, Brian M. Wolpin, Rachael Z. Stolzenberg-Solomon, Alison P. Klein, Laufey T. Amundadottir, Ann L. Oberg

**Affiliations:** 1 Department of Quantitative Health Sciences, Mayo Clinic, Jacksonville, FL USA; 2 Department of Quantitative Health Sciences, Mayo Clinic, Rochester, MN USA; 3 Department of Medicine, Mayo Clinic, Rochester, MN USA; 4 Laboratory of Translational Genomics, Division of Cancer Epidemiology and Genetics, National Cancer Institute, National Institutes of Health, Bethesda, MD USA; 5 Department of Oncology, Sidney Kimmel Comprehensive Cancer Center, Johns Hopkins School of Medicine, Baltimore, MD USA; 6 Department of Obstetrics and Gynecology, New York University Grossman School of Medicine, New York, NY USA; 7 Department of Population Health, New York University Grossman School of Medicine, New York, NY USA; 8 Perlmutter Cancer Center, NYU Langone Health, New York, NY USA; 9 Division of Cancer Epidemiology and Genetics, National Cancer Institute, National Institutes of Health, Bethesda, MD USA; 10 Department of Epidemiology and Biostatistics, University of California, San Francisco, San Francisco, CA USA; 11 Genomic Epidemiology Group, German Cancer Research Center (DKFZ), Heidelberg, Germany; 12 Paris-Saclay University, UVSQ, Inserm, Gustave Roussy, CESP, Villejuif, France; 13 Nuffield Department of Population Health, Oxford University, Oxford, UK; 14 Department of Epidemiology and Biostatistics, Memorial Sloan Kettering Cancer Center, New York, NY USA; 15 Lunenfeld-Tanenbaum Research Institute, Sinai Health System and University of Toronto, Toronto, ON Canada; 16 SWOG Statistical Center, Fred Hutchinson Cancer Center, Seattle, WA USA; 17 Division of Cancer Epidemiology, German Cancer Research Center (DKFZ), Heidelberg, Germany; 18 Department of Biology, University of Pisa, Pisa, PI Italy; 19 Division of Public Health Sciences, Fred Hutchinson Cancer Center, Seattle, WA USA; 20 Cancer Epidemiology Program, University of Hawaii Cancer Center, Honolulu, HI USA; 21 Population Health Program, QIMR Berghofer, Brisbane, QLD Australia; 22 School of Public Health, University of Queensland, Brisbane, QLD Australia; 23 Department of Population Science, American Cancer Society, Atlanta, GA USA; 24 University at Buffalo School of Public Health and Health Professions, Buffalo, NY USA; 25 Genomic Epidemiology Branch, International Agency for Research on Cancer, Lyon, France; 26 Division of Epidemiology, Department of Medicine, Vanderbilt Epidemiology Center, Vanderbilt-Ingram Cancer Center, Vanderbilt University School of Medicine, Nashville, TN USA; 27 Department of Epidemiology, Johns Hopkins School of Public Health, Baltimore, MD USA; 28 Division of Research, Kaiser Permanente Northern California, Pleasanton, CA USA; 29 New York University Grossman School of Medicine, New York, NY USA; 30 Nutrition and Metabolism Branch, International Agency for Research on Cancer, World Health Organization, Lyon, France; 31 Department of Epidemiology, Harvard T.H. Chan School of Public Health, Boston, MA USA; 32 Department of Nutrition, Harvard T.H. Chan School of Public Health, Boston, MA USA; 33 Department of Pathology, Sol Goldman Pancreatic Cancer Research Center, Johns Hopkins School of Medicine, Baltimore, MD USA; 34 Department of Preventive Medicine, Keck School of Medicine, University of Southern California, Los Angeles, CA USA; 35 Department of Gastrointestinal Medical Oncology, University of Texas MD Anderson Cancer Center, Houston, TX USA; 36 Genetic and Molecular Epidemiology Group, Spanish National Cancer Research Centre, Madrid, Spain; 37 Department of Medical Oncology, Dana-Farber Cancer Institute, Boston, MA USA; 38 CIBER Epidemiologíay Salud Pública (CIBERESP), Barcelona, Spain; 39 Hospital del Mar Institute of Medical Research (IMIM), Universitat Autònoma de Barcelona, Barcelona, Spain; 40 Escuela Andaluza de Salud Pública (EASP), Granada, Spain; 41 Instituto de Investigación Biosanitaria ibs.GRANADA, Granada, Spain; 42 Centro de Investigación Biomédica en Red de Epidemiología y Salud Pública (CIBERESP), Madrid, Spain; 43 Division of Preventive Medicine, Brigham and Women’s Hospital, Boston, MA USA; 44 Precision Medicine, School of Clinical Sciences at Monash Health, Monash University, Clayton, VIC Australia; 45 Cancer Epidemiology Division, Cancer Council Victoria, Melbourne, VIC Australia; 46 Centre for Epidemiology and Biostatistics, Melbourne School of Population and Global Health, The University of Melbourne, Melbourne, VIC Australia; 47 Mayo Clinic, Rochester, MN USA; 48 Regeneron Genetics Center, Tarrytown, NY USA; 49 Department of Chronic Disease Epidemiology, Yale School of Public Health, New Haven, CT USA

**Keywords:** Cancer, Genetics, Oncology

## Abstract

Polygenic risk scores (PRSs) may enhance risk stratification for pancreatic ductal adenocarcinoma (PDAC), but existing models vary widely in design, predictive performance, and cross-ancestry transferability. We developed genome-wide PRSs using Bayesian methods (LDpred2 and PRS-CS) and *p* value thresholding (PRSice-2) and systematically evaluated these alongside 13 published PRSs to identify models with robust predictive performance across ancestries. Using GWAS summary statistics from 7531 cases and 10,631 controls, we derived the PRSs and tested associations in an independent sample of 4508 PDAC cases and 46,189 controls, with adjustment for well-established PDAC risk factors. Among all models, the genome-wide LDpred2-based PRS showed the strongest association with PDAC (OR = 1.57 per standard deviation increase; 95% CI: 1.51–1.62) and significantly improved discrimination beyond established risk factors alone (AUC = 0.74–0.76; *p* < 0.0001). Importantly, the genome-wide LDpred2 PRS demonstrated consistent associations across African, Admixed American, and European ancestry groups, whereas the best-performing published PRS was associated with PDAC risk only in individuals of European ancestry. These findings support genome-wide PRSs as a promising framework for multi-ancestry risk stratification for PDAC and to inform targeted early detection strategies.

## Introduction

Pancreatic ductal adenocarcinoma (PDAC) is the third leading cause of cancer-related death in the U.S. and is projected to become the second by 2030^1,2^. Globally, PDAC ranks 12th in cancer incidence but 6th in cancer mortality, underscoring its disproportionate lethality^3^. More than 80% of patients present with locally advanced or metastatic disease, contributing to a poor overall 5-year survival rate of 8%^1^. However, the 5-year survival is 3% for metastatic disease compared with 44% for localized tumors, highlighting the critical importance of early detection^1^. Despite this need, the low prevalence of PDAC in the general population makes population-wide screening impractical, as reflected in the 2019 U.S. Preventive Services Task Force recommendation against PDAC screening for average-risk individuals^4^. In contrast, targeted screening of moderate-to-high-risk individuals may offer a more effective approach to early detection^5–7^.

Individuals with a first-degree family history of PDAC have an over twofold higher risk of developing PDAC, with the risk rising further as the number of affected relatives increases, underscoring the contribution of hereditary factors^8,9^. However, the genetic basis of PDAC is dominated by somatic driver mutations, most notably in *KRAS*, *TP53*, *CDKN2A*, and *SMAD4*, that play major roles in tumorigenesis^10–14^. In addition, rare pathogenic germline variants in genes such as *ATM*, *BRCA1/2*, *CDKN2A*, *PALB2*, and *TP53* confer moderate-to-high risk and account for approximately 5–10% of cases^15–18^. Common genetic variation also contributes to PDAC susceptibility, but with modest risk magnitudes. Genome-wide association studies (GWASs) have thus far identified 31 PDAC risk loci, including 22 in European ancestry populations (PanScan I-III and PanC4)^19–24^, five in a Chinese population^25^, and four in Japanese populations^26,27^. An additional locus in the *TERT–CLPTM1L* region was identified through a candidate gene approach by the PANDoRA consortium^28,29^. Notably, there is limited overlap between susceptibility loci identified in the European and East Asian populations, suggesting ancestry-specific genetic architecture underlying risk^22,23,30^.

Although GWASs have advanced our understanding of the genetic etiology of PDAC, the individual risk SNPs identified to date have modest effect sizes^19–24^. Aggregating these variants into polygenic risk scores (PRSs) may improve risk stratification and help identify moderate-to-high-risk individuals for targeted surveillance and clinical management^6,31^. At present, at least 13 PRS models have been developed for PDAC^23,32–44^, but they vary widely in design, including differences in SNP selection, which limits comparability. Restricting existing PRSs to genome-wide significant SNPs also excludes variants with smaller effects that may nonetheless enhance predictive performance. Further, because most PDAC GWASs were conducted in European and East Asian populations, PRSs derived from these studies may perform suboptimally in other ancestry groups.

Other PRS modeling approaches, such as LDpred2^45^ and PRS-CS^46^, apply Bayesian shrinkage methods to adjust SNP effect sizes based on genome-wide linkage disequilibrium (LD) structure and can incorporate large numbers of variants with modest effects to enhance risk prediction. PRSice-2^47^, which uses a *p* value thresholding approach, may also enhance risk modeling. These approaches have been used to improve risk modeling for colorectal, breast, and prostate cancers^48,49^ and might similarly improve PRS-based risk stratification for PDAC.

The aims of this study were twofold: first, to validate existing published PDAC PRS models in an independent cohort using the same SNPs employed in each study; and second, to compare these models with newly developed genome-wide PRSs generated using LDpred2 and PRS-CS, and the *p* value thresholding PRSice-2 to identify the optimal approach for risk prediction. We hypothesize that genome-wide PRSs will outperform previously published models, providing improved risk stratification and greater potential to inform early detection strategies.

## Results

### Participant characteristics and risk factor distribution

Distributions of demographic characteristics and PDAC risk factors among the Mayo Clinic cases and controls are shown in Table 1. The PDAC cases were on average six years older than controls and included a higher proportion of men. The cases were also more likely than controls to report a family history of pancreatic cancer, heavy alcohol use, current smoking, and a history of type II diabetes, and they had a higher prevalence of chronic pancreatitis.

**Table 1:** Characteristics of the study participants (*N* = 50,697)

Characteristics	PDAC cases (*n* = 4508)	Controls (*n* = 46,189)
Age at consent		
Mean (SD)	65.7 (10.4)	60.4 (15.2)
Range	21 - 92	18 - 99
BMI at enrollment		
Mean (SD)	28.4 (5.7)	28.8 (6.0)
Range	12.7 - 64.0	11.6 - 73.2
Missing	22	398
Sex		
Male	2568 (57.0%)	19,457 (42.1%)
Female	1940 (43.0%)	26,732 (57.9%)
Family history of pancreatic cancer^a^		
Yes	392 (9.7%)	1768 (4.2%)
No	3660 (90.3%)	40342 (95.8%)
Missing	456	4079
Heavy alcohol use^b^		
Yes	465 (15.2%)	1476 (3.3%)
No	2599 (84.8%)	43231 (96.7%)
Missing	1444	1482
Chronic pancreatitis		
Yes	395 (8.8%)	88 (0.2%)
No	4113 (91.2%)	46101 (99.8%)
ABO blood group		
O	2921 (64.8%)	26647 (57.7%)
Non-O	1585 (35.2%)	19528 (42.3%)
Missing	2	14
Smoking history		
Never	2042 (46.3%)	26468 (57.9%)
Former	1758 (39.8%)	17046 (37.3%)
Current	615 (13.9%)	2210 (4.8%)
Missing	93	465
Type 2 diabetes mellitus		
Yes	1273 (33.3%)	8318 (18.0%)
No	2551 (66.7%)	37871 (82.0%)
Missing	684	0
Genetic ancestry		
African	65 (1.4%)	647 (1.4%)
Admixed American	61 (1.4%)	417 (0.9%)
East Asian	30 (0.7%)	334 (0.7%)
European	4334 (96.1%)	44406 (96.1%)
South Asian	11 (0.2%)	200 (0.4%)
Unassigned	7 (0.2%)	185 (0.4%)

*BMI* body mass index, *PDAC* pancreatic ductal adenocarcinoma, *SD* standard deviation.

^a^Having two or more first-degree relatives diagnosed with any pancreatic cancer type.

^b^Consuming ≥ 3 drinks of alcohol per day or having a history of alcohol abuse disorder.

### Risk factor associations with PDAC

Associations between each covariate and PDAC risk are provided for both the base and full models (Table 2). In the full model, nearly all risk factors were associated with higher PDAC risk, except former smoking, which showed no association, and self-reported BMI, which showed an inverse association. The strongest risk factor was chronic pancreatitis, with affected individuals having over 40-fold higher odds of PDAC (OR = 43.35, 95% CI: 33.92–55.40, *p* < 0.0001).

**Table 2:** . Associations of variables in the base and full models with risk of PDAC

	Base model^a^		Full model^b^	
Characteristics	OR (95% CI)	*P* value	OR (95% CI)	*P* value
Age, years	1.02 (1.02,1.03)	5.93 ×10^-91^	1.03 (1.03,1.03)	3.92 × 10^–100^
Sex, male	1.63 (1.54,1.74)	3.69 ×10^-53^	1.42 (1.33,1.52)	1.15 × 10^–23^
Self-reported BMI, kg/m^2,c^			0.97 (0.97,0.98)	9.42 × 10^–16^
Family history of pancreatic cancer^d^			2.18 (1.92,2.48)	6.50 × 10^-31^
Heavy alcohol Use^e^			4.41 (3.84,5.06)	6.86 × 10^–53^
Chronic pancreatitis			43.35 (33.92,55.40)	2.12 × 10^–197^
Non-O ABO blood group			1.34 (1.25,1.43)	6.13 × 10^–17^
Smoking, Former			0.95 (0.89,1.03)	0.20
Smoking, Current			3.00 (2.67,3.37)	1.27 × 10^–72^
Type 2 diabetes mellitus			1.95 (1.8,2.11)	3.69 × 10^–55^

*BMI* body mass index, *CI* conidence interval, *PC* principal components, *PDAC* pancreatic ductal adenocarcinoma, *OR* odds ratio.

^a^Based model included age (continuous), sex, and top 10 principal components, assessed simultaneously.

^b^Full model included the base model variables plus BMI (continuous), family history of pancreatic cancer (yes vs. no), heavy alcohol use (yes vs. no), chronic pancreatitis (yes vs. no), ABO blood group (non-O vs. O), smoking history (former, current, vs. never), and type 2 diabetes mellitus (yes vs. no).

^c^BMI was calculated based on self-reported usual adult weight and height for cases, and self-reported weight and height at recruitment for controls.

^d^History of any pancreatic cancer type in at least two first-degree relatives.

^e^Consuming ≥ 3 drinks of alcohol per day or having a history of alcohol abuse disorder.

### PRS effect sizes and discriminatory performance

**Fig. 1:**
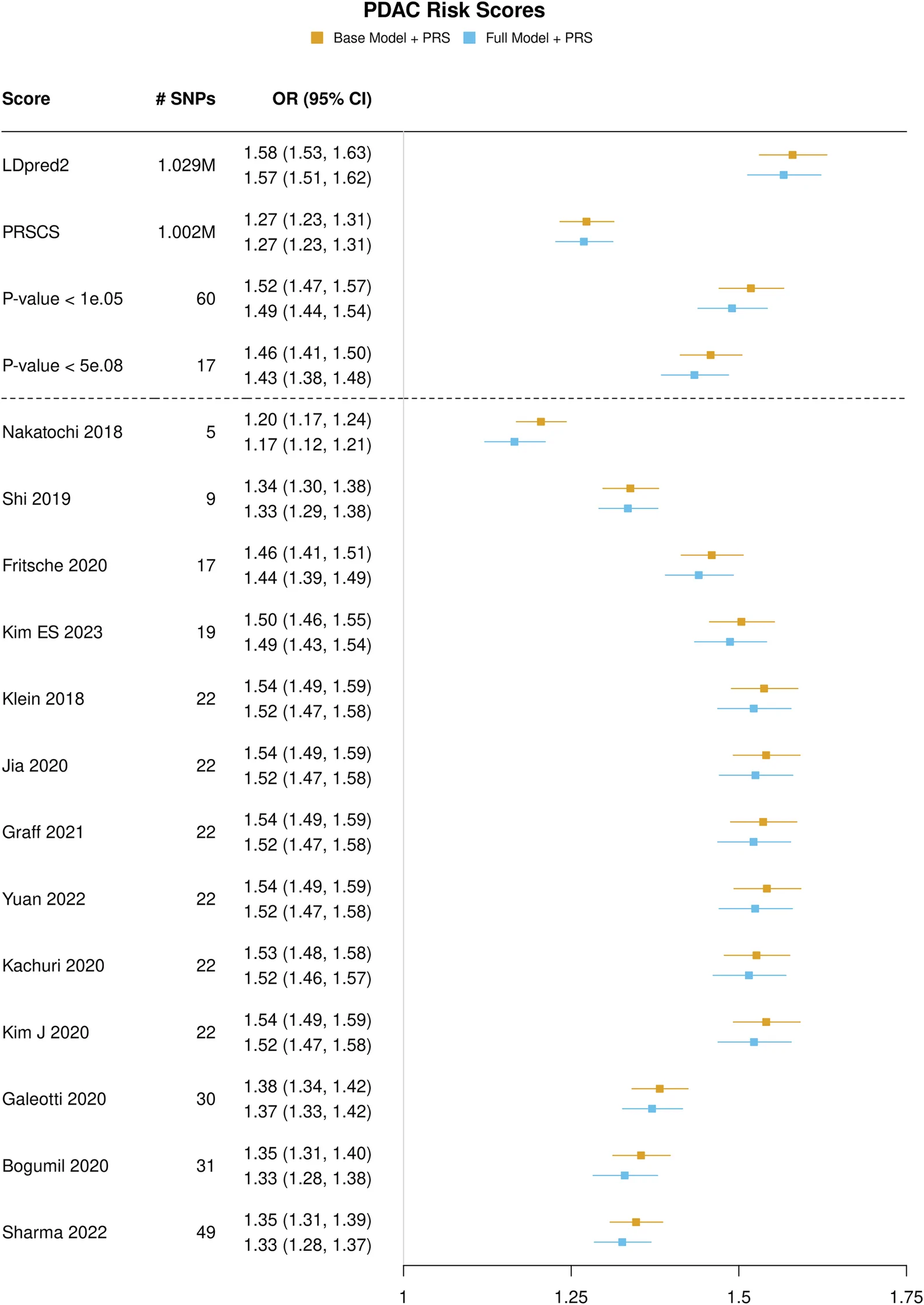
Associations of newly constructed Bayesian-based polygenic risk scores (PRS) and previously published models with pancreatic ductal adenocarcinoma (PDAC) risk. The X-axis shows odds ratios (ORs) and 95% confidence intervals (CIs) per one standard deviation increase in the PRS. Bayesian models estimated using the leave-Mayo-out GWAS are displayed above the black horizontal line, and previously published models are shown below. LDpred2 and PRS-CS employ Bayesian frameworks to infer the optimal number of SNPs to include, whereas the other PRSs are based on fixed genome-wide significance thresholds (*p* < 5 × 10⁻^08^) or a less stringent *p* value (<1 × 10⁻^05^). The **base model** + **PRS** (yellow color) evaluates the association between the PRS and PDAC risk, adjusting for age (continuous), sex, and the top 10 ancestry principal components (continuous). The **full model** + **PRS** (blue color) includes additional adjustment for body mass index (continuous), first-degree family history of pancreatic cancer (yes/no), heavy alcohol use (yes/no), chronic pancreatitis (yes/no), ABO blood group (O/non-O), smoking history (never/former/current), and diabetes mellitus (yes/no). The PRSs by Nakatochi et al. (2018) and Bogumil et al. (2020) were derived from Japanese and multi-ethnic populations, respectively. The rest were based on European ancestry populations. CI confidence interval, M million, OR odds ratio, PDAC pancreatic ductal adenocarcinoma.

Effect sizes of standardized PDAC PRS models generated using LDpred2, PRS-CS, and PRSice-2 (with *p* value thresholds of 1 × 10⁻⁵ and 5 × 10⁻⁸), and previously published PDAC PRSs, were compared (Fig. 1). PRS effect sizes were estimated after adjustment for age, sex, and the top 10 PCs (base model) or for all covariates (full model). Among these, the LDpred2-based PRS showed the largest effect size in the full model (OR = 1.57, 95% CI: 1.51–1.62, *p* < 0.0001). The second most strongly associated PRSs, based on 22 GWAS-identified SNPs (e.g., Klein et al., 2018), showed a slightly smaller effect size (OR = 1.52, 95% CI: 1.47–1.58, *p* < 0.0001). However, the corresponding AUCs for the LDpred2 PRS (AUC = 0.76, 95% CI: 0.75–0.77) and the Klein et al. PRS (AUC = 0.76, 95% CI: 0.75–0.77) did not differ significantly by DeLong’s test (*p* = 0.12). Notably, the PRS constructed using only genome-wide significant variants (*p* < 5×10⁻⁸) from our training GWAS had a smaller effect size (OR = 1.43, 95% CI: 1.38–1.48, *p* < 0.0001).

**Fig. 2:**
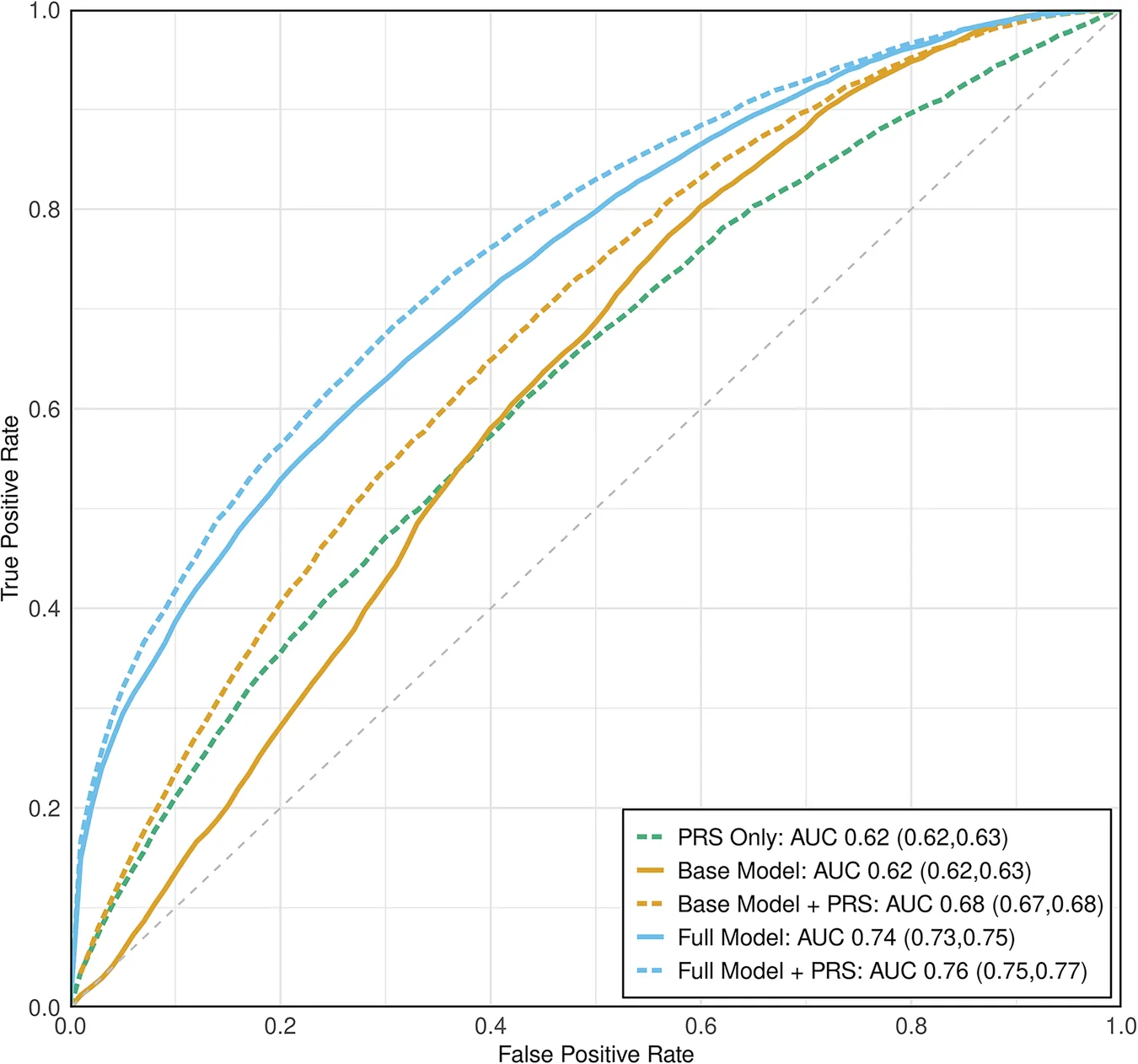
Area under the receiver operating characteristic (AUC) curves for prediction of pancreatic adenocarcinoma risk. **PRS only** (dashed green line) comprised the LDpred2 polygenic risk score (PRS, continuous) and top 10 principal components (continuous). **Base Model** (solid yellow line) comprised age (continuous), sex, and the top 10 principal components of genetic ancestry (continuous). **Base Model** + **PRS** (dashed yellow line) comprised variables in the base model and the LDpred2 polygenic risk score. **Full Model** (solid blue line) comprised age (continuous), sex, top 10 principal components (continuous), body mass index (continuous), first-degree family history of pancreatic cancer (yes, no), heavy alcohol use (yes, no), chronic pancreatitis (yes, no), ABO blood group (O, non-O), smoking history (never, former, current), and type 2 diabetes mellitus (yes, no). **Full Model** + **PRS** (dashed blue line) included all variables in the full model and the LDpred2 polygenic risk score.

Model discrimination based on AUCs for the base covariate-only model was 0.62 (95% CI: 0.62–0.63), which increased to 0.74 (95% CI: 0.73–0.75) in the full covariate-only model (Fig. 2). Adding the LDpred2 PRS to the base model significantly improved discrimination, increasing the AUC from 0.62 (95% CI: 0.62–0.63) to 0.68 (95% CI: 0.67–0.68); DeLong’s test for difference between models *p* < 0.0001. Similarly, adding the LDpred2 PRS to the full model further improved discrimination, increasing the AUC from 0.74 (95% CI: 0.73–0.75) to 0.76 (95% CI: 0.75–0.77); DeLong’s test for difference *p* < 0.0001.

We next assessed PRS effect sizes after dichotomizing the PRSs at a potential clinical decision-making cutpoint to define a “high PDAC risk” (Supplementary Table 2). Individuals above the 95th percentile of the LDpred2-based PRS had 2.52-fold higher odds of PDAC (OR = 2.52, 95% CI: 2.26–2.81, *p* < 0.0001) compared with those below the 95th percentile, based on distribution among controls. This threshold identified 12% (n = 545) of PDAC cases in the top 5% of the LDpred2 PRS.

### Cross-ancestry evaluation of SNP PRS models

**Fig. 3:**
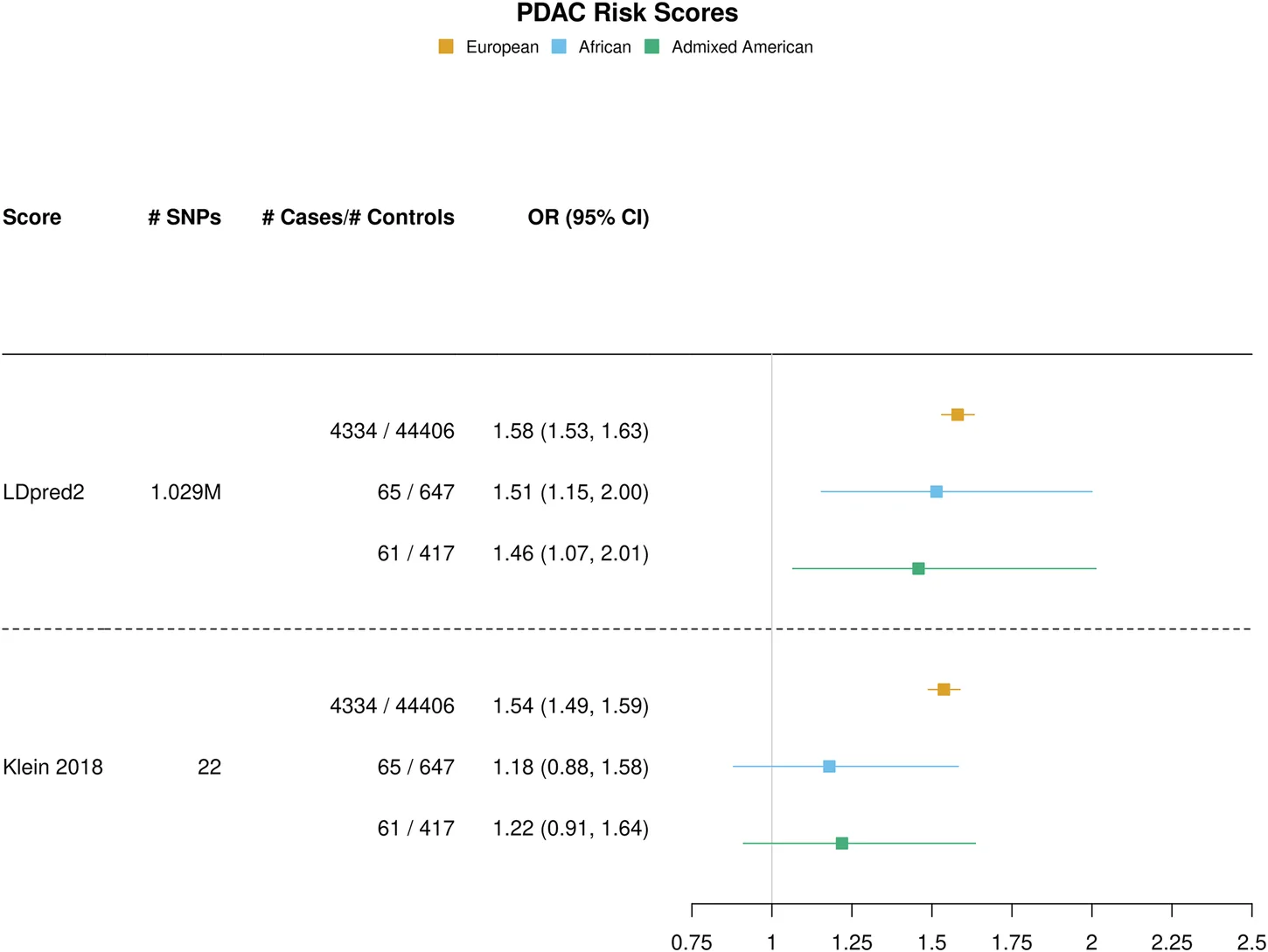
Associations of a newly constructed Bayesian-based LDpred2 polygenic risk score (PRS) and a previously published PRS (Klein et al., 2018) with risk of pancreatic ductal adenocarcinoma (PDAC), stratified by genetic ancestry (European, African, and Admixed American). The X-axis displays odds ratios (ORs) and 95% confidence intervals (CIs) per one standard deviation increase in the PRS. Colored bars denote ancestry groups: yellow for European, blue for African, and green for Admixed American participants. Ancestry-specific normalization was performed, adjusting scores using ancestry-specific mean and standard deviations.

Finally, we evaluated the cross-ancestry performance of two PRSs: a genome-wide model (LDpred2; based on 1.029 million SNPs) and a candidate SNP model (Klein et al., 2018; 22 SNPs), stratified by genetic ancestry with ancestry-specific normalization (Fig. 3). The Klein et al. PRS showed limited transferability, with non-significant associations in both African (OR = 1.18, 95% CI: 0.88–1.58, *p* = 0.27) and Admixed American (OR = 1.22, 95% CI: 0.91–1.64, *p* = 0.18) samples, but with a significant association in European samples (OR = 1.54, 95% CI: 1.49–1.59, *p* < 0.0001). In contrast, the LDpred2 PRS showed a pattern of consistent associations across ancestries: African (OR = 1.51, 95% CI: 1.15–2.00, *p* = 0.003), Admixed American (OR = 1.46, 95% CI: 1.07–2.01, *p* = 0.018), and European (OR = 1.58, 95% CI: 1.53–1.63, *p* < 0.0001). Tests for heterogeneity supported cross-ancestry consistency for LDpred2 (Cochran’s Q = 0.34, *p* = 0.84) and suggested a trend toward heterogeneity for the Klein et al. 2018 PRS (Q = 5.51, *p* = 0.06), indicating that genome-wide PRS models may provide more stable performance across diverse ancestries than candidate SNP-based models.

## Discussion

We evaluated the performance of previously published PRS models for PDAC in an independent sample using the same SNP sets as in the original studies and applied consistent covariate adjustments across models to enable direct comparison. In parallel, we developed a new genome-wide PRS for PDAC using LDpred2 and PRS-CS and the *p* value thresholding PRSice-2, adjusting for established PDAC risk factors. Under comparable conditions, the genome-wide LDpred2-based PRS model (1.029 million SNPs) had a slightly higher effect size, but the AUC was similar to that of the best performing previously published models that used 22 GWAS-significant SNPs. In the fully adjusted model, each standard deviation increase in the LDpred2 PRS was associated with 1.57-fold higher odds of PDAC, and individuals in the highest 5% of the PRS distribution had 2.52-fold higher odds of PDAC compared with the remaining 95% of participants. AUCs for a full model with non-genetic risk factors only, a model with genetic factors only (LDpred2 PRS and the top 10 PCs), and a model with both genetic and non-genetic factors were 0.74, 0.62, and 0.76, respectively. Notably, the fully adjusted LDpred2 PRS model identified 12% of PDAC cases within the highest 5% of the PRS distribution in controls, considered having a high polygenic risk for PDAC. The LDpred2-based PRS also demonstrated far greater consistency across ancestry groups compared with the best-performing published model^23^.

PDAC is a rapidly fatal malignancy, and its early detection remains an intractable challenge^50,51^ but can be improved with better risk stratification to identify individuals at elevated risk among whom cancer surveillance and screening can be implemented to improve outcomes^52,53^. Although studies of rare hereditary risk variants for PDAC have translated into clinical genetic testing, a critical barrier is that there is far less known for the ≥90% of patients who do not carry rare germline pathogenic variants in cancer predisposition genes^15,19,54,55^. A viable PRS model for PDAC could be an important adjunct to early detection efforts. However, the potential translation of PRSs to clinically useful tools to identify individuals at elevated PDAC risk has progressed slowly.

Here, we evaluated existing PDAC PRSs alongside newly developed models to identify the best-performing approach for risk stratification. A full covariate-based model demonstrated good discrimination (AUC = 0.74) and could be readily applicable in clinical settings. Adding the PRS yielded a modest but statistically significant improvement (AUC = 0.76). Thus, established risk factors alone provide substantial predictive value, while the incremental benefit of PRS should be interpreted within a multifactorial risk framework. At present, the modest gain in performance does not clearly justify clinical implementation of the genome-wide LDpred2-based PRS beyond the covariate-only model, particularly considering cost and feasibility. However, further refinement through iterative incorporation of additional risk markers and external validation may enhance performance and clinical utility. As genotyping becomes more integrated into clinical practice, PRS-based models may thus have increased relevance in the future. Further, our LDpred2-based PRS showed improved cross-ancestry transferability, particularly among individuals of African and Admixed American ancestries, even after ancestry-specific normalization. However, given the limited sample sizes in these groups, these findings should be interpreted with caution and require validation in larger, more diverse cohorts.

In our study population, the LDpred2-based PRS outperformed the PRS-CS model despite both being genome-wide approaches. It is known that the relative performance of genome-wide PRS approaches depends on factors such as the LD reference panel, variant set, hyperparameter tuning, and the underlying genetic architecture of the trait. LDpred2 and PRS-CS rely on different Bayesian priors and tuning strategies: LDpred2 tunes parameters such as polygenicity and SNP heritability, with an optional sparse formulation, whereas PRS-CS uses a continuous-shrinkage prior with either tuned or automatically learned global shrinkage^45,46^. Accordingly, our findings suggest that, under our analytic conditions, LDpred2 provided a better fit for PDAC risk prediction than PRS-CS.

Previous PDAC PRSs vary widely in design, SNP selection, and covariate adjustment, limiting direct comparison^23,32–44^. Early models based on GWAS-significant SNPs reported about 2–3-fold higher PDAC risk for individuals in the highest PRS strata^23,36,41,43^, though many did not adjust for established PDAC risk factors^23,36^ or may be affected by sample overlap bias^41^. Further, most were based on summary statistics from the same GWAS (Klein et al. 2018), including its own PRS^23^, and they all had similar effect sizes and some had overlapping samples. To address these issues, we evaluated existing PRSs on a unified scale and compared them with genome-wide approaches. The genome-wide LDpred2-based PRS showed slightly higher effect size but similar discrimination to the Klein et al. PRS^23^. Furthermore, despite potential effect size inflation due to sample overlap, the Klein et al. PRS still showed a smaller effect than the LDpred2 PRS, which was derived without such overlap. Also, LDpred2 demonstrated more consistent performance across non-European ancestries than the Klein et al. PRS, suggesting potential advantages for cross-ancestry generalization for genome-wide approaches, though this requires verification in other studies.

Key strengths of our study include the large sample size and careful clinical phenotyping of the PDAC cases through individual medical record review by trained abstractors, supervised by subspecialty physicians. Using LDpred2 to derive the PRS allowed us to incorporate latent risk SNPs beyond GWAS-significant variants, expanding on previous PRSs that were largely restricted to genome-wide significant SNPs. We also adjusted for all well-established clinical and lifestyle risk factors for PDAC, reflecting relevant effects under likely clinical risk modeling. The use of an ultra-rapid case-finding process ensured that approximately 86% of our PDAC cases were recruited within 30 days of diagnosis, with an overall high case response rate (68%), minimizing the potential impact of survival bias^56,57^. Additionally, we performed validation analyses of previously published PRS models in an independent sample by recalculating risk estimates for GWAS-significant SNPs identified in the PanScan and PanC4 GWASs after excluding Mayo Clinic samples and using the Mayo samples for independent assessment of the previously published models.

Limitations of our study include our predominantly European ancestry population (96%), although this didn’t seem to limit the performance of the LDpred2 model in minority populations. A limitation of the PRS model is that it may reflect multiple underlying pathways or mechanisms that influence PDAC risk that may not be easily detectable for targeting prevention or interception. Further, we did not perform an exhaustive benchmarking of all available genome-wide PRS methods. While no single approach has yet demonstrated consistent superiority across cancers^58–60^, it remains possible that alternative methods could have higher predictive performance for PDAC risk. Because of the relative infrequency of PDAC, perhaps more than large numbers of genetic risk variants or few common risk factors might be needed to detect appreciable risk magnitude to enhance risk stratification for this cancer^44,61^. The modifiable risk factors included in our model, such as smoking, BMI, and alcohol use, were measured at a single time point and thus may not reflect cumulative lifetime exposure. Additionally, BMI was self-reported, limiting granularity, and may explain the inverse association observed between BMI and PDAC in this study. The retrospective case-control design also introduces the possibility of recall bias in reporting prior exposures. Our design did not allow for direct estimation of absolute risk, which should be addressed in future prospective cohorts. Also, our study cohort was drawn from a tertiary clinic with high referral rates and might not be a perfect representation of the general population.

In summary, we evaluated previously published PRS models for PDAC and developed a new genome-wide PRS using a Bayesian modeling framework (LDpred2). Under comparable conditions, the LDpred2-based PRS demonstrated a slightly higher effect size, but a similar AUC compared with the second best-performing published model. Notably, the LDpred2 PRS exhibited greater consistency across ancestries. Further refinement of the model could enhance risk stratification, enabling targeted clinical surveillance and screening for moderate-to-high-risk individuals and ultimately supporting earlier detection of this highly lethal cancer across ancestry groups.

## Methods

### Study population

Genetic and risk factor data were obtained from patients recruited into the Mayo Clinic Biospecimen Resource for Pancreas Research registry between October 2000 and October 2020 who had a pathologically or radiographically confirmed diagnosis of incident PDAC^56,57,62^. Genetic and risk factor data on control patients recruited from primary care clinics at Mayo Clinic between April 2009 and March 2016 and who did not have a history of pancreatic cancer were obtained from the Mayo Clinic Biobank^63^. Participation rates were 68% for the Mayo Clinic Biospecimen Resource for Pancreas Research registry and 21% for the Mayo Clinic Biobank. Details on the study design and recruitment procedures have been published previously^56,57,63^. The Mayo Clinic Biospecimen Resource for Pancreas Research registry and the Mayo Clinic Biobank used similar risk factor questionnaires to solicit information on participant demographics, usual adult weight and height, alcohol use, smoking history, and type II diabetes. We calculated body mass index (BMI, kg/m^2^) based on self-reported usual adult weight and height, and abstracted data on chronic pancreatitis from individual medical records. Participants’ ABO blood group was genetically determined as non-O versus O using two well-established ABO SNPs (rs505922/rs687289 and rs8176746), as described previously^57^. We also obtained previously published summary statistics and individual-level GWAS data from the PanScan and PanC4 consortia on PDAC cases and controls^19–24,64^. This study was conducted in accordance with the ethical principles of the Declaration of Helsinki and the Declaration of Istanbul. All participants provided written informed consent, including consent for the use of their biospecimens and data in future research studies. The study protocol was approved by the Mayo Clinic Institutional Review Board (IRB #22-001158; approval date June 5, 2023).

### Genotyping

Peripheral blood DNA from Mayo Clinic PDAC cases and controls was sequenced by the Regeneron Genetics Center (RGC, Tarrytown, NY) using a custom capture panel as part of the Mayo Clinic Project Generation initiative. The panel incorporated exome regions and common variant coverage through a genotyping-by-sequencing approach^65,66^. Downstream processing and quality control (QC) procedures included variants exclusion if they failed to meet minimum thresholds for call rate ( < 95%), minor allele frequency (MAF, <0.5%), or deviation from Hardy-Weinberg equilibrium (*p* < 1 × 10⁻⁶). Sample-level exclusions were made for individuals exhibiting high rates of missing genotypes ( > 5%), sex discrepancies, or evidence of aberrant heterozygosity ( < 70% across multiple chromosomes)^66^. Relatedness filtering was conducted through iterative analysis with PLINK and PRIMUS, identifying and removing individuals with excess kinship (PI_HAT > 0.1875 with >100 relatives or PI_HAT > 0.08 with >25,000 relatives)^66^. When a second-degree or closer relationship was observed between cases and controls, we retained the case and excluded the control(s). When two cases or two controls had a second-degree or closer relationship, the one with more complete covariate data was retained. Imputation was performed via the TOPMed Imputation Server^67,68^, and only variants with high imputation quality (dosage R² >0.8) and MAF ≥ 5% were retained. After QC, data from 4508 PDAC cases and 46,189 controls remained for the final analysis **(**Supplementary Table 1).

### Leave-Mayo-out GWAS analysis

Because Mayo Clinic samples contributed to the GWASs used in developing many of the published PRS models (through inclusion in PanScan and PanC4), potential bias could arise from sample overlap influencing SNP selection and effect size estimates. To address this, we leveraged data from a previously published PanScan I-III and PanC4 GWAS, as described by Klein et al.^23^, to generate SNP weights after excluding Mayo Clinic participants. Each genotyping array was re-imputed using the TOPMed Imputation Server to ensure consistency across datasets. Mayo samples (1509 cases and 1865 controls) were excluded for use in an independent validation cohort, resulting in a reanalyzed dataset of 7531 cases and 10,631 controls for updating SNP weights. GWAS analysis was then re-run using PLINK2, adjusting for the same covariates as in the original analysis (i.e., study site, geographic region, age, sex, and ancestry principal components [PCs])^23^. Per-array summary statistics were combined using a fixed-effects inverse-variance meta-analysis in METAL^69^. Supplementary Figure 1 shows Manhattan plots comparing the PanScan I-III and PanC4 GWAS results and those obtained after excluding Mayo Clinic participants.

### Published PRS models for PDAC

We manually curated published PDAC PRS models from the literature^23,32,41,42,70^ and the Polygenic Score (PGS) Catalog^71,72^. Four palindromic SNPs were identified and replaced with proxy SNPs in high linkage disequilibrium (r² > 0.98). Details of each model, including the number of SNPs used, and the source GWAS from which SNPs were derived, are provided in Supplementary Table 2. The overlap of SNPs across models and information on proxy SNPs used to replace those found to be palindromic are also provided in Supplementary Fig. 2.

### PRSs for PDAC

We estimated PRSs for PDAC in the Mayo Clinic sample using GWAS summary statistics derived from the reanalyzed PanScan I-III and PanC4 datasets. Three complementary approaches were applied. First, we implemented LDpred2-auto^73^, a Bayesian shrinkage method that adjusts SNP effect sizes based on genome-wide LD structure using the HapMap3+ European LD reference panel, automatically estimating key model parameters without requiring external tuning samples. Second, we used PRS-CS-auto^69^, another Bayesian shrinkage method that employs a continuous shrinkage prior to infer SNP weights, optimized with the 1000 Genomes Project European LD reference panel provided in the PRS-CS GitHub. Finally, we applied PRSice-2^47^ using the traditional clumping-and-thresholding method, with *p* value thresholds of 1 × 10^–5^ and 5 × 10^–8^ and default clumping parameters.

### Imputation of covariate data

To account for missing values in covariates (Supplementary Fig. 3), we performed multiple imputation by chained equations using the *mice* package in the R software^42^ incorporating the entire set of covariates (age, BMI, sex, family history of pancreatic cancer, alcohol use, chronic pancreatitis, ABO blood group, smoking status, type II diabetes), PRS, and case-control status in the imputation model. Following iterative chained-equation modeling, 30 imputed datasets were generated.

### Statistical analyses

We fit the following logistic regression models treating case-control status as the outcome: (1) a **base model** that included age, sex, and top 10 ancestry PCs; (2) a **base model** + **PRS** that added each PRS of interest separately to the base model; (3) a **full model** that added the following covariates to the base model: BMI, family history of pancreatic cancer, alcohol use, chronic pancreatitis, ABO blood group, smoking status, and type II diabetes; and (4) a **full model** + **PRS** that added each PRS to the full model. This approach allowed comparisons between PRS-only, covariates-only, and integrated models to determine whether the PRS improves prediction beyond what can be predicted with covariates alone. Logistic regression model results were reported as odds ratios (ORs) with 95% confidence intervals (CIs). Each model was fitted to each imputed dataset, and results were pooled using Rubin’s rules to account for uncertainty induced by missingness. Model discrimination was assessed using the area under the receiver operating characteristic curve (AUC), and final estimates were pooled as log-transformed values across multiple imputations. We compared the performance of the published PRS models with novel PRS models estimated using LDpred2, PRSCS, and PRSice-2. To assess the potential for clinical decision-making, we assessed risk for individuals in the top five percent of the PRS, determined based on the distribution of the PRS in controls only. To compare association of the PRS across ancestries, we repeated the models above within each genetically determined ancestry (European, African, and Admixed American) having effective sample size (4 × *p* × [1 – *p*] × *N*, where *p* is the sample prevalence) greater than 100. For these analyses, we performed ancestry-specific normalization, adjusting scores using ancestry-specific mean and standard deviation. All statistical analyses were performed using R v.4.4.1 (R CORE Team, Vienna, Austria).

## Supplementary information

Supplementary information

## Data Availability

The Pancreatic Cancer Cohort Consortium (PanScan) and Pancreatic Cancer Case–Control Consortium (PanC4) GWAS data used to develop the polygenic risk scores, have been deposited in dbGaP (accession numbers phs000206.v5.p3 and phs000648.v1.p1, respectively). Individual-level genetic data from the Project Generation cohort used for validation analyses are subject to contractual restrictions and are not publicly available. These data were generated by the Regeneron Genetics Center as part of a collaboration with Mayo Clinic and may be accessed only by approved investigators within secure Mayo Clinic computing environments, in accordance with institutional and data use agreements. Summary-level results supporting the findings of this study for the validation analyses are available from the corresponding author upon reasonable request, subject to institutional and contractual approvals. Code Availability: The underlying code for this study is not publicly available but may be made available to qualified researchers on reasonable request to the corresponding author.

## Code availability

The underlying code for this study is not publicly available but may be made available to qualified researchers on reasonable request to the corresponding author.
