# Supplementary material for: Polygenic scores for risk of pancreatic ductal adenocarcinoma: evaluation of novel and published models
Source: NPJ Precis Oncol. 2026 May 27;10:324. doi: 10.1038/s41698-026-01479-x (PMC13490364; doi:10.1038/s41698-026-01479-x)
Supplement: Supplementary file 1 [file 41698_2026_1479_MOESM1_ESM.pdf]

Supplementary Material

Supplementary Table 1. Sequential Sample QC steps.		
	Cases (N=4568)	Controls (N=52,606)
Sex discordance	12	48
Poor Exome coverage	5	12
Contamination > 5%	9	23
Duplicates	1	42
Controls with PDAC	0	277
Related pairs	33	6015
Final Analytic Sample	4508	46189

Supplementary Table 2. Details of previously published PRS models for pancreatic cancer from the polygenic scores (PGS) catalog.

Author (year)	Number of SNPs	Samples in PRS development	Cases/controls	SNP selection method	PGS catalog reference	Summary statistics source[a]
Nakatochi 2018[1]	5	Multi-institutional Japanese case-control study and HERPACC (Aichi Cancer Center)	942/2074	Screening analysis of 61 previously identified SNPs using a p-value of 0.05 and stepwise forward selection.	N/A	Multi-institutional Japanese case-control study and HERPACC (Aichi Cancer Center) (PMID: 30192808)
Shi 2019[2]	9	PanScan I-III, and PanC4.<br><br>Evaluated in TCGA (cases) and eMERGE (controls; includes Mayo patients)	163/13427	Previously published risk SNPs.	PGS000159	Summary statistics from Klein et al. (2018; PMID: 29422604) were used by these authors.
Fritsche 2020[3]	17	Michigan Genomics Initiative and UKBB	430/4292, 659/6590	GWAS hits, LD Clumping (MAF ≥ 1%, $r^2 \leq 0.1$) & *p*-value ≤ $5e^{-08}$	PGS000385	Used GWAS results from GWAS catalog.
Kim ES (2023)[4]	19	UKBB	149 Female, 182 male/<br><br>44610 Female/38403 male	Fixed threshold p<5e-8, LD clumping (r2 ≤ 0.1)	PGS004250	Summary statistics from Klein et al. (2018; PMID: 29422604) were used by these authors.
Kachuri (2020)[5]	22	UKBB	493/390998	GWAS significant hits *p*-value < 5e08, MAF > 1%, biallelic SNPs, LD-thin $r^2 > 0.3$	PGS000794	Summary statistics from Klein et al. (2018; PMID: 29422604) were used by these authors.

Supplementary Table 2 (continued).

Author (years)	Number of SNPs	Samples in PRS development	Cases/controls	SNP selection method	PGS catalog reference	Summary statistics source[a]
Kim J (2020)[6]	22	PanScan I-III, PanC4. Evaluated in Health Professionals Follow-up Study, Nurses' Health STudy, Physician's Health Study, Women's Health Initiative Observational Study	500/1091	Klein *et al.* 2018 (PMID: 29422604)	PGS000663	Summary statistics from Klein et al. (2018; PMID: 29422604) were used by these authors.
Klein 2018[7]	22	PanScan I-III, PanC4	9040/12496	22 independent genome-wide significant risk SNPs	N/A	GWAS using PanScan I-III, and PanC4 (PMID: 29422604)
Jia 2020[8]	22	PanScan I-III, and PanC4. Evaluated in UKBB	432/400812 total patients used. 14,584 have one of the cancers evaluated but it is not clear whether non-pancreatic cancers were included in the control group for pancreatic cancer	22 GWAS hits from Klein *et al*. 2018 paper	PGS000725	Summary statistics from Klein et al. (2018; PMID: 29422604) were used by these authors.
Graff 2021[9]	22	GERA, UKBB	665/410354	GWAS significant hits *p*-value < 5e08, MAF > 1%, biallelic SNPs, LD-thin $r^2 > 0.3$	PGS000083	Used GWAS results from 7 GWAS including the study by Klein et al. 2018 (PMID: 29422604)
Yuan 2022[10]	22	PanScan I-III, PanC4	5107/8845	22 GWAS hits from Klein 2018 paper	PGS002740	Summary statistics from Klein et al. (2018; PMID: 29422604) were used by these authors.

Supplementary Table 2 (continued).

	Number of SNPs	Samples in PRS development	Cases/controls	SNP selection method	PGS catalog reference	Summary statistics source[a]
Galeotti (2020)[11]	30	PANDora	839/2040	Known risk SNPs (p<5x10-8, 18 SNPs, p<10-7, 11 SNPs) + ABO SNPs	N/A	Summary statistics from Klein et al. (2018; PMID: 29422604) were used by these authors.
Bogumil 2020[12]	31	Multiethnic Cohort and the Southern Community Cohort Study	691/13778	22 GWAS significant SNPs from Klein et. Al., 2018 plus additional GWAS hits from multi-ethnic studies	N/A	Weights calculated in the MEC and SCCS cohorts (PMID: 32958499)
Sharma 2022[13]	49	UKBB	1042/10420	SNPs from previous GWAS	N/A	Largest weight from Nakatochi (includes SNPs from Klein 2018, Galeotti et al. 2020 (PanScan I-III/PanC4), Jia (Klein 2018), Rashkin (UKBB and GERA), Molina (GWAS catalog including PanScan I-III and PanC4 GWAS. Additional details are provided in PMID: 35065983.

[a]All original summary statistics included Mayo data, except those from Nakatochi et al. (2018) and Bogumil et al. (2020). For the present study, however, SNP weights were re-estimated after excluding Mayo samples (see main manuscript for details).

1. Nakatochi M, Lin Y, Ito H, Hara K, Kinoshita F, Kobayashi Y, et al. Prediction model for pancreatic cancer risk in the general Japanese population. PLoS One. 2018;13:e0203386.
2. Shi Z, Yu H, Wu Y, Lin X, Bao Q, Jia H, et al. Systematic evaluation of cancer-specific genetic risk score for 11 types of cancer in The Cancer Genome Atlas and Electronic Medical Records and Genomics cohorts. Cancer Med. 2019;8:3196-205.
3. Fritsche LG, Patil S, Beesley LJ, VandeHaar P, Salvatore M, Ma Y, et al. Cancer PRSweb: An Online Repository with Polygenic Risk Scores for Major Cancer Traits and Their Evaluation in Two Independent Biobanks. Am J Hum Genet. 2020;107:815-36.
4. Kim ES, Scharpf RB, Garcia-Closas M, Visvanathan K, Velculescu VE, Chatterjee N. Potential utility of risk stratification for multicancer screening with liquid biopsy tests. NPJ Precis Oncol. 2023;7:39.

5. Kachuri L, Graff RE, Smith-Byrne K, Meyers TJ, Rashkin SR, Ziv E, et al. Pan-cancer analysis demonstrates that integrating polygenic risk scores with modifiable risk factors improves risk prediction. Nat Commun. 2020;11:6084.
6. Kim J, Yuan C, Babic A, Bao Y, Clish CB, Pollak MN, et al. Genetic and Circulating Biomarker Data Improve Risk Prediction for Pancreatic Cancer in the General Population. Cancer Epidemiol Biomarkers Prev. 2020;29:999-1008.
7. Klein AP, Wolpin BM, Risch HA, Stolzenberg-Solomon RZ, Mocci E, Zhang M, et al. Genome-wide meta-analysis identifies five new susceptibility loci for pancreatic cancer. Nat Commun. 2018;9:556.
8. Jia G, Lu Y, Wen W, Long J, Liu Y, Tao R, et al. Evaluating the Utility of Polygenic Risk Scores in Identifying High-Risk Individuals for Eight Common Cancers. JNCI Cancer Spectr. 2020;4:pkaa021.
9. Graff RE, Cavazos TB, Thai KK, Kachuri L, Rashkin SR, Hoffman JD, et al. Cross-cancer evaluation of polygenic risk scores for 16 cancer types in two large cohorts. Nat Commun. 2021;12:970.
10. Yuan C, Kim J, Wang QL, Lee AA, Babic A, Amundadottir LT, et al. The age-dependent association of risk factors with pancreatic cancer. Ann Oncol. 2022;33:693-701.
11. Galeotti AA, Gentiluomo M, Rizzato C, Obazee O, Neoptolemos JP, Pasquali C, et al. Polygenic and multifactorial scores for pancreatic ductal adenocarcinoma risk prediction. J Med Genet. 2021;58:369-77.
12. Bogumil D, Conti DV, Sheng X, Xia L, Shu XO, Pandol SJ, et al. Replication and Genetic Risk Score Analysis for Pancreatic Cancer in a Diverse Multiethnic Population. Cancer Epidemiol Biomarkers Prev. 2020;29:2686-92.
13. Sharma S, Tapper WJ, Collins A, Hamady ZZR. Predicting Pancreatic Cancer in the UK Biobank Cohort Using Polygenic Risk Scores and Diabetes Mellitus. Gastroenterology. 2022;162:1665-74.e2.

Abbreviations: GERA, Genetic Epidemiology Research on Adult Health and Aging; GWAS, genome-wide significant study; LD, linkage disequilibrium; MAF, minor allele frequency; PanC4, Pancreatic Cancer Case Control Consortium; PanScan, Pancreatic Cancer Cohort Consortium; SNPs, single nucleotide polymorphisms; UKBB, the UK Biobank.

Supplementary Table 3. Associations of the various polygenic risk scores with PDAC risk using categorical variables, comparing the top five percent to the bottom 95%, and to the average risk individuals (middle 40th – 60th percentile distribution in controls).

PRS	# SNPs	PDAC cases in top 5% [a] N (%)	Top 5% versus bottom 95% [a,b] OR (95% CI)	P-value	Top 5% versus middle 40th- 60th %ile [a,b] OR (95% CI)	P-value
LDpred2	1.029M	545 (12.09)	2.52 (2.26,2.81)	6.24×10^{-77}	1.88 (1.68,2.11)	1.99×10^{-25}
PRSCS	1.002M	370 (8.21)	1.66 (1.47,1.88)	6.75×10^{-22}	1.37 (1.21,1.56)	2.83×10^{-06}
P-value <1e05	60	529 (11.73)	2.4 (2.15,2.68)	9.58×10^{-58}	1.82 (1.62,2.04)	9.14×10^{-23}
P-value <5e08	17	508 (11.27)	2.26 (2.02,2.53)	1.44×10^{-53}	1.77 (1.58,2)	1.61×10^{-20}
Nakatochi 2018	5	325 (7.21)	1.33 (1.17,1.52)	1.81×10^{-05}	1.20 (1.05,1.38)	9.63×10^{-03}
Shi 2019	9	442 (9.8)	2.14 (1.91,2.41)	2.55×10^{-35}	1.75 (1.55,1.98)	4.05×10^{-18}
Fritsche 2020 (17 SNPs)	17	499 (11.07)	2.21 (1.98,2.47)	1.29×10^{-39}	1.80 (1.60,2.02)	1.63×10^{-21}
Kim ES 2023	19	509 (11.29)	2.22 (1.99,2.49)	8.60×10^{-54}	1.75 (1.56,1.97)	9.56×10^{-20}
Klein 2018	22	526 (11.67)	2.31 (2.07,2.58)	2.06×10^{-579}	1.74 (1.55,1.96)	6.03×10^{-20}
Jia 2020	22	521 (11.56)	2.28 (2.04,2.55)	1.86×10^{-61}	1.73 (1.54,1.94)	1.74×10^{-19}
Graff 2021	22	523 (11.6)	2.32 (2.08,2.59)	9.08×10^{-61}	1.78 (1.59,2.00)	2.19×10^{-21}
Yuan 2022	22	531 (11.78)	2.31 (2.07,2.58)	1.39×10^{-58}	1.76 (1.57,1.98)	8.12×10^{-21}
Kachuri 2020	22	519 (11.51)	2.31 (2.06,2.58)	4.39×10^{-56}	1.78 (1.59,2.00)	3.25×10^{-21}
Kim J 2020	22	530 (11.76)	2.31 (2.07,2.58)	1.10×10^{-56}	1.76 (1.57,1.98)	6.11×10^{-21}
Galeotti 2020	30	410 (9.09)	2.27 (2.02,2.56)	7.62×10^{-35}	1.84 (1.63,2.09)	2.58×10^{-18}
Bogumil 2020	31	395 (8.76)	1.68 (1.49,1.9)	3.54×10^{-25}	1.41 (1.24,1.6)	3.93×10^{-07}
Sharma 2022	49	374 (8.3)	1.70 (1.51,1.93)	1.15×10^{-31}	1.35 (1.19,1.54)	6.92×10^{-06}

[a]Cut-points for the top 5% and middle 40th % to 60th % were determined based on distributions among controls only.
[b]All models adjusted for age (continuous), sex, top 10 ancestry principal components (continuous), body mass index (continuous), first-degree family history of pancreatic cancer (yes, no), heavy alcohol use (yes, no), chronic pancreatitis (yes, no), ABO blood group (O, non-O), smoking history (never, former, current), and type 2 diabetes mellitus (yes, no).

A.

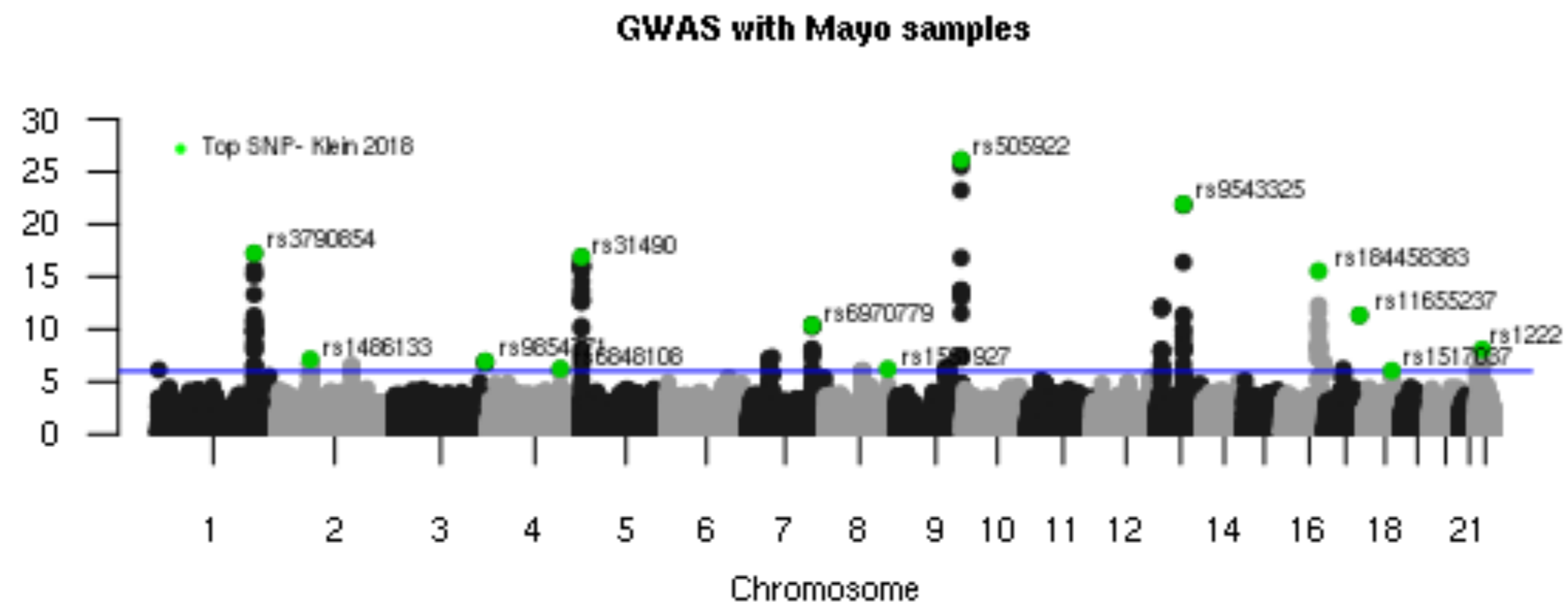
GWAS with Mayo samples
Top SNP- Klein 2018
-log10(p)
rs3790854
rs1486133
rs31490
rs6970779
rs505922
rs9543325
rs184458383
rs11655237
Chromosome

B.

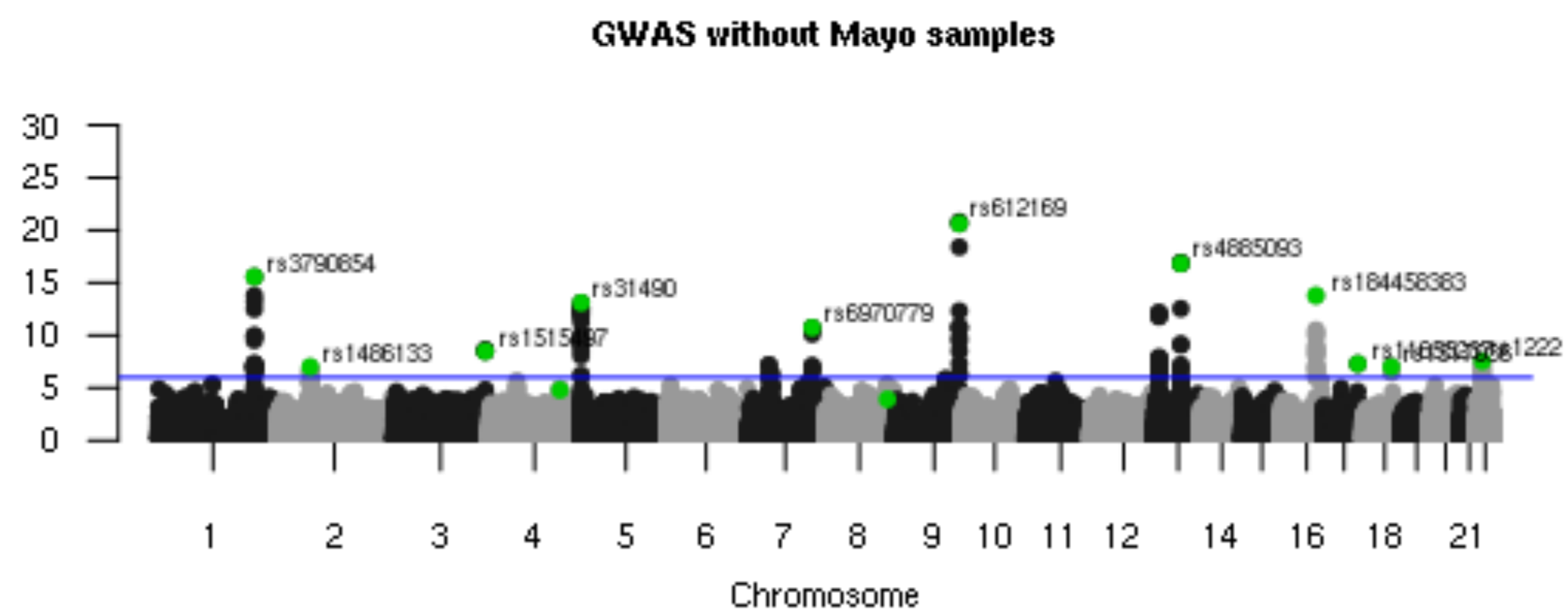
GWAS without Mayo samples
-log10(p)
rs3790854
rs1486133
rs31490
rs6970779
rs612169
rs4885093
rs184458383
Chromosome

C.

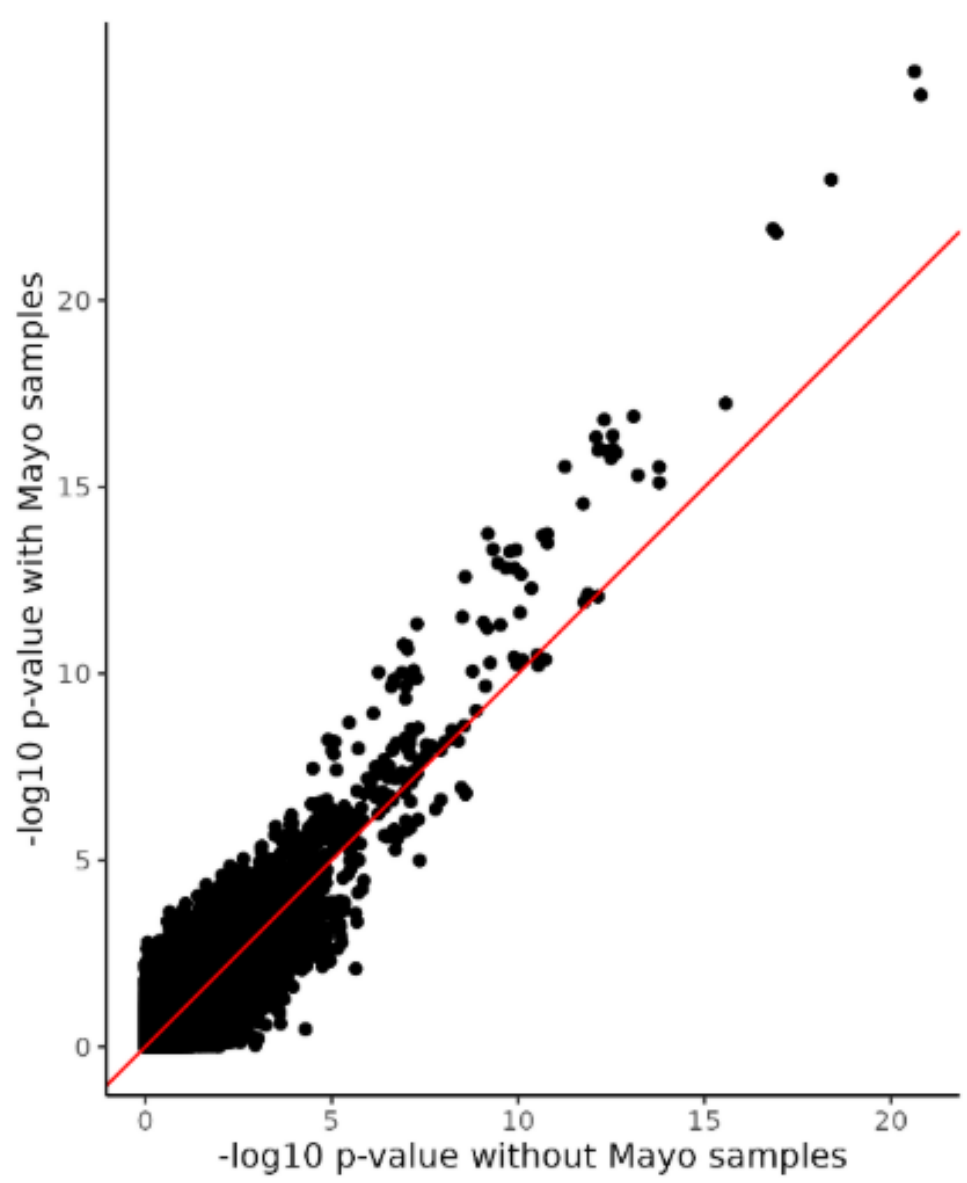
-log10 p-value with Mayo samples
-log10 p-value without Mayo samples

Supplementary Figure 1. Comparison of genome-wide association study (GWAS) results for pancreatic ductal adenocarcinoma (PDAC). (A) Manhattan plot for the full PanScan and PanC4 GWAS including all participants. (B) Manhattan plot for the PanScan and PanC4 GWAS excluding Mayo Clinic participants ("leave-Mayo-out" analysis). (C) Scatter plot comparing $-\log_{10}(p)$ values from the two analyses, with the red line indicating the line of identity.

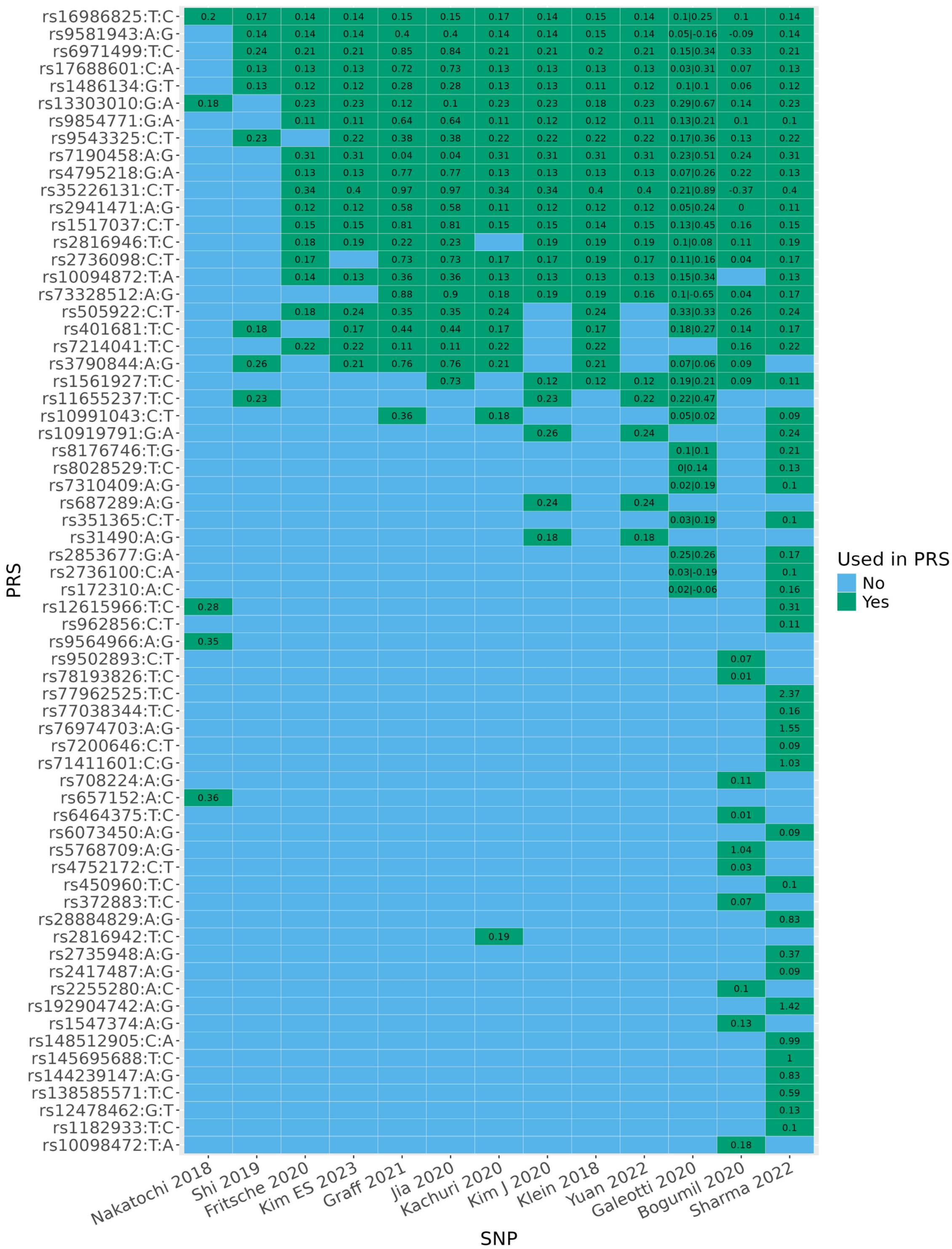

Supplementary Figure 2. Heatmap showing the overlap of SNPs across polygenic score (PGS) models. The numbers within cells represent the SNP weights used in each PRS model. For the model by *Galeotti et al.* (2020), separate weights are shown for homozygous and heterozygous risk genotypes, respectively. SNPs rs2816946, rs73328512, and rs4752172 serve as proxy variants for corresponding loci in the original model(s).

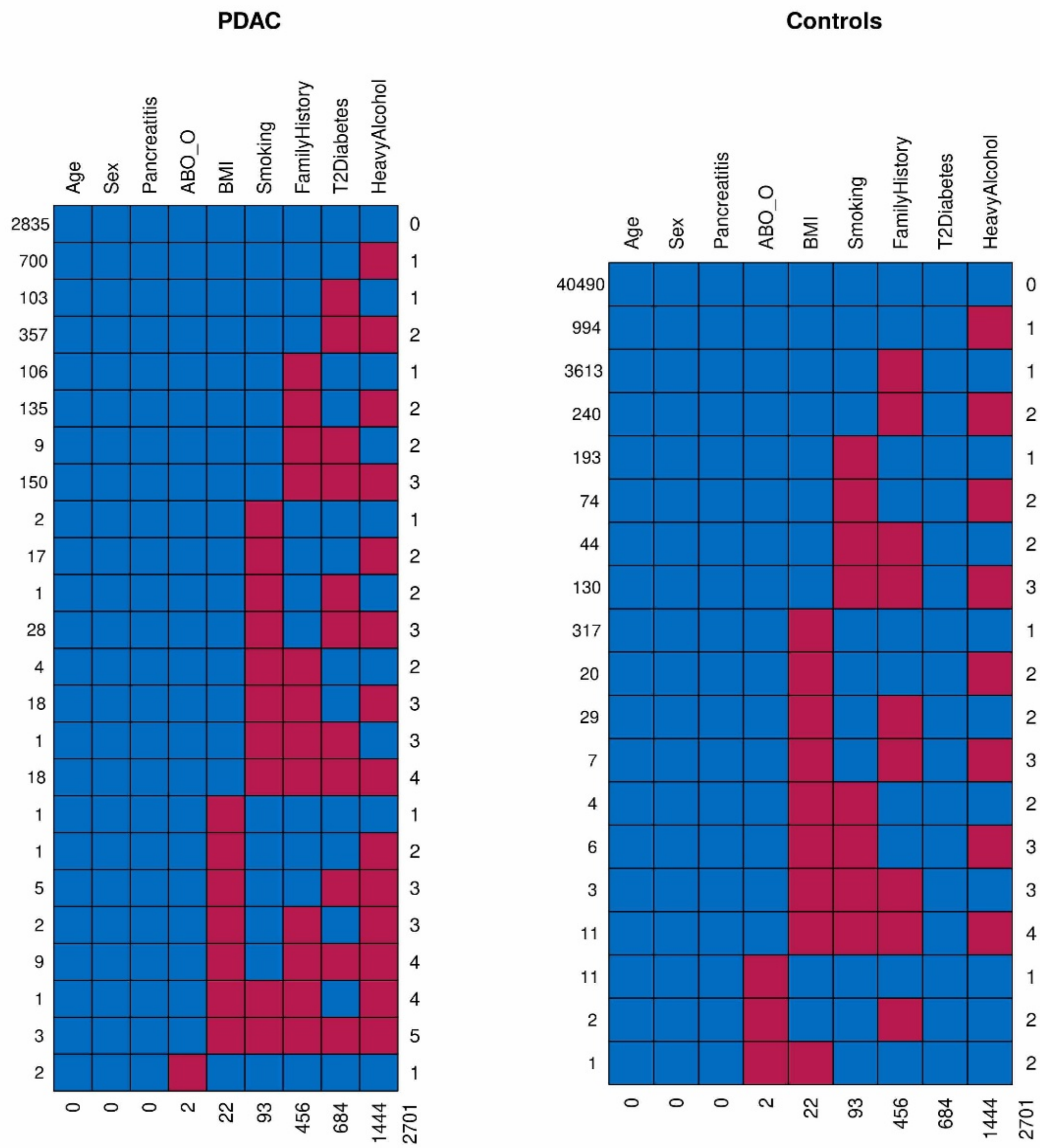

Supplementary Figure 3. Heatmap depicting completeness of epidemiologic covariates for pancreatic ductal adenocarcinoma cases (left) and controls (right). Blue cells indicate complete data, and pink cells indicate missing data. The top axis lists covariate names. The left axis shows the number of individuals corresponding to each missingness pattern, while the bottom axis displays the number of individuals missing the variable in that row. The right axis indicates the number of variables missing within each missingness pattern.
